# Cell-Based Strategies for Meniscus Tissue Engineering

**DOI:** 10.1155/2016/4717184

**Published:** 2016-05-05

**Authors:** Wei Niu, Weimin Guo, Shufeng Han, Yun Zhu, Shuyun Liu, Quanyi Guo

**Affiliations:** ^1^Beijing Key Laboratory of Regenerative Medicine in Orthopaedics, Key Laboratory of Musculoskeletal Trauma & War Injuries, PLA, Institute of Orthopaedics, Chinese PLA General Hospital, 28 Fuxing Road, Haidian District, Beijing 100853, China; ^2^First Hospital of Shanxi Medical University, Shanxi Medical University, No. 65, Jiefang Nan Road, Yingze District, Taiyuan 030012, China; ^3^Institute of Orthopedics, First Hospital of Shanxi Medical University, No. 85, Jiefang Nan Road, Yingze District, Taiyuan 030012, China

## Abstract

Meniscus injuries remain a significant challenge due to the poor healing potential of the inner avascular zone. Following a series of studies and clinical trials, tissue engineering is considered a promising prospect for meniscus repair and regeneration. As one of the key factors in tissue engineering, cells are believed to be highly beneficial in generating bionic meniscus structures to replace injured ones in patients. Therefore, cell-based strategies for meniscus tissue engineering play a fundamental role in meniscal regeneration. According to current studies, the main cell-based strategies for meniscus tissue engineering are single cell type strategies; cell coculture strategies also were applied to meniscus tissue engineering. Likewise, on the one side, the zonal recapitulation strategies based on mimicking meniscal differing cells and internal architectures have received wide attentions. On the other side, cell self-assembling strategies without any scaffolds may be a better way to build a bionic meniscus. In this review, we primarily discuss cell seeds for meniscus tissue engineering and their application strategies. We also discuss recent advances and achievements in meniscus repair experiments that further improve our understanding of meniscus tissue engineering.

## 1. Introduction

As a worldwide medical problem, treatment of meniscus injuries has long been a research focus [1–6]. In adult, the distribution of meniscus neurovascular is complex with heterogeneity [7]. The outside region (red-red zone) is full of neurovascular tissues, while the inside region (white-white zone) lacks neurovascular tissue, and the region (red-white zone) between the former two areas displays a transitional characteristic from both the red-red and white-white regions. The neurovascular distribution is often associated with the prognosis of patients with meniscus tear. It is usually difficult to repair the injuries in the white-white zone [8]. The meniscus performs important functions in load bearing, shock absorption, joint lubrication, and joint stability [9–13]. Injury to this structure can greatly influence joint motion and daily living [14, 15]. According to one report, the incidence of meniscus injury resulting in meniscectomy is 61/100,000, of which the medial meniscus represents 81% and the lateral meniscus, 19% [1]. And it will be higher in athletes [16–18].

Meniscectomy is an effective way to relieve pain and joint swelling. However, follow-up surveys show that this type of surgery may damage knee stability and accelerate the development of osteoarthritis, so that many patients undergoing meniscus resection eventually have to accept a total knee arthroplasty [19–22]. Therefore, this choice may be beneficial for short-term purposes but may cause more long-term damage. An arthroscopic partial meniscectomy may be a better method to reduce joint damage [23, 24]. To maintain the stability of the knee, the surgeon may perform a minimally invasive arthroscopic meniscorrhaphy to repair a lacerated meniscus, which results in improved function compared to a meniscectomy and allows earlier joint motion [25–27]. In young patients, to better protect articular cartilage and restore knee function, an “ideal” solution would be meniscus replacement or regeneration [28]. Meniscal allograft transplantation (MAT) can improve knee function and result in good clinical outcomes; however, further evidence is necessary to determine whether it is chondroprotective [29, 30]. Furthermore, it is difficult to resolve resource, shape matching, and ethical issues. Tissue engineering using natural or synthetic matrices as a scaffold to guide tissue repair or regeneration in three dimensions shows promising prospects for meniscus regeneration [31].

In meniscus tissue engineering, a scaffold is the basis for regenerating a new structure. Scaffold materials are typically selected from polymeric synthetic materials, such as polyglycolic acid (PGA) and poly-L-lactic acid (PLA), and natural biological products, such as silk, collagen type I, and proteoglycans [32–35]. The production process has ranged from “traditional” molding to electrospinning and more recently to three-dimensional (3D) printing technology [32, 33, 36, 37]. Research regarding scaffold preparation has made great progress. Scientists at Columbia University successfully built a meniscus with polycaprolactone (PCL) via 3D printing technology, which was loaded with connective tissue growth factor (CTGF) and transforming growth factor- (TGF-) *β*3. It was shown to induce internal stem cell migration and differentiation to regenerate a new meniscus [37]. This treatment has been successful in sheep. Stone et al. [38, 39] developed a collagen type I scaffold isolated from bovine tendon that has been used clinically. Growth factors such as TGF and fibroblast growth factors (FGF) play important roles in regulating cell growth and cell differentiation and are regarded as key elements in tissue engineering [40–42]. The growth factors were also used to induce cells to differentiate from stem cells to obtain more cell resources for meniscus tissue engineering [34, 43–45]. Moreover, the studies also revealed that a hypoxic environment is able to slow down cell dedifferentiation process, and mechanical stimulation can improve collagen and glycosaminoglycan (GAG) secretion [44, 46–48]. A bioreactor, which simulates the knee microenvironment, can provide a better platform for meniscus tissue engineering research [49, 50].

The study has shown that a cell-based tissue-engineered meniscus achieves better repair results than cell-free ones [51]. Therefore, current research on meniscus tissue engineering has focused on the use of cells. As one of the three elements of tissue engineering, the choice of cell seed is particularly important. In early research, cells used in meniscus tissue engineering were mainly of a single cell type, and it was difficult to copy the native tissue features. Inspired by the coexistence of cells in the knee and their interaction, researchers carried out cell cocultures to build a tissue-engineered meniscus with better mechanical and cartilaginous properties. This method also solved cell supply issues to some extent. After further understanding the complex structure of the meniscus, combined with the theory of cell coculture, zonal recapitulation was proposed to mimic the organizational heterogeneity of the meniscus. To increase cell interactions and reduce the interference of nonbiological components, research was also conducted to build a tissue-engineered meniscus with cell self-assembly alone without any scaffolds. In this review, we review the reports on cell-based meniscus repair and discuss cell application strategies from single cell type, cell coculture, zonal recapitulation, and scaffold-free cell self-assembly, respectively (Table 1).

## 2. Single Cell Type with a Scaffold

As a “classical” strategy for a tissue-engineered meniscus, this method has been used in many studies and as the basis for further cell-based developments. The cells, which include fibrochondrocytes, chondrocytes, synoviocytes, or stem cells, are all involved in building a construct with scaffolds. Herein, we discuss the application of these cells one by one to find an appropriate seed for meniscus tissue engineering.

## 3. Meniscus Fibrochondrocytes

**Figure 1:**
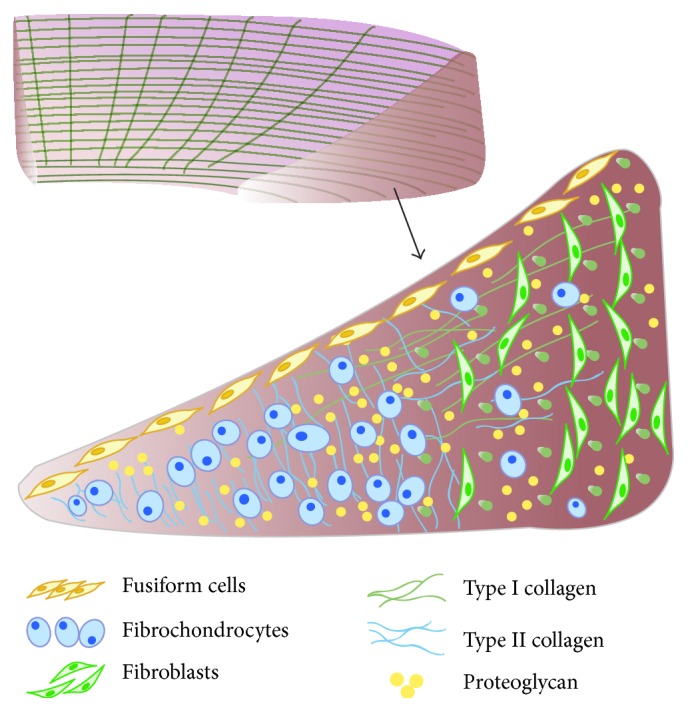
Schematic diagram of meniscus internal ultrastructure. Most of collagen fibers bundles oriented circumferentially; only few of them presented a radial alignment. Cells of the superficial zone are fusiform in shape and located below the tissue surface; the cells in the outer one-third region or vascular zone are mainly elongated fibroblasts, and collagen I accounts for >90%; for inner two-thirds region or avascular zone, round or oval shaped fibrochondrocytes are interspersed, the ratio of collagen I and collagen II is about 2 : 3.

The structure of the meniscus is complex with heterogeneity (Figure 1). Meniscal extracellular matrix (ECM) mainly comprised collagen fibers and proteoglycans. Most of collagen fibers bundles oriented circumferentially; only few of them presented a radial alignment. The oriented structure of the fibrillar collagens is closely associated with meniscus biomechanical properties. Therefore, it is important to restore the microarchitecture of the collagen fibers for successfully repairing or regenerating the meniscus [52]. The populations of meniscus cells can be divided into three distinct groups: fibrochondrocytes are cells with a round or oval shape mostly located in the inner two-thirds of the meniscus; they express both collagen I and collagen II. Fibroblast-like cells are elongated and found mainly in the outer one-third, the ECM of which is mainly collagen I. Cells of the superficial zone are fusiform in shape and located below the tissue surface [53–55].

Currently, most studies focusing on cells isolated from the meniscus involve fibrochondrocytes [56, 57]. One advantage of a substitute built with in situ derived cells is better histocompatibility. Baker et al. [58] conducted an experiment in which meniscus cells isolated from 10 human donors who underwent knee surgery were expanded to passage two in monolayer culture and then seeded onto PCL fiber-aligned biodegradable nanofibrous scaffolds and cultured for 10 weeks. The results showed that all constructs seeded with meniscus cells increased in dry weight, DNA, collagen, and GAG contents in a time-dependent manner, and the mechanical properties of the meniscus-derived cell-laden constructs strongly correlated with collagen content, but not donor age. Correlation analysis between collagen content in constructs and the mechanical properties suggests that increasing the collagen deposition of constructs may further enhance their mechanical properties. Similarly, this indicated that autologous cells derived from the patients themselves could represent a potential cell resource for meniscus tissue engineering. However, the number of primary cells isolated from the meniscus was not sufficient to satisfy the needs for tissue engineering. To overcome this, an approach often used is to expand primary cells to passage two or three in monolayer culture until the cell number is sufficient. However, cellular phenotypes and gene expression levels can be changed during passaging, indicating that cells are in a dedifferentiation process [59]. Other studies have shown that the addition of fibroblast growth factor (FGF) into the culture solution or as a coating on the scaffold can inhibit cell dedifferentiation and promote secretion of the ECM [41, 60, 61]. Culturing cells in hypoxic conditions also have a similar effect [46, 47]. Kang et al. [32] used fibrochondrocytes seeded on PGA/PLGA scaffolds to repair rabbit models with a total meniscectomy in vivo. After 6 and 10 weeks, a neomeniscus formed with the appearance of fibrocartilaginous tissue. Moreover, the histological and immunohistochemical structures in the neomeniscus were similar to those of the native meniscus. After 36-week transplantation, histochemical and immunohistochemical structures of neomeniscus were more aligned than those of 10 weeks. Similarly, in the anterior areas, biomechanical properties in neomeniscus were higher than those of native meniscus. However, biomechanical properties of neomeniscus at middle and posterior sites were lower than those of native meniscus. Esposito et al. [62] preseeded fibrochondrocytes on PLDLA/PCL-T scaffolds for three weeks, at which point the constructs were implanted to replace the medial meniscus in rabbits. The constructs showed good compatibility with surrounding tissues, and fibrocartilaginous tissue with mature collagen fibers appeared in histology results after 24 weeks. These experiments indicate the feasibility of regenerating meniscus with meniscus cells using tissue engineering.

## 4. Articular Chondrocytes

The limited supply of allogeneic or autologous meniscus cells limits their application for meniscus tissue engineering, and monolayer expansion can lead to significant changes in cellular phenotype [59]. Cartilaginous cells, such as articular chondrocytes, are regarded as a promising cell source. Fibrochondrocytes and chondrocytes are both from cartilage tissue, and a study showed their similar cell membrane markers and high expression of collagen II, the major component of the ECM [55]. These similarities in cellular phenotype and molecular biology make it possible for chondrocytes to be used as cell seeds for meniscus tissue engineering. Yoo et al. [63] embedded PLGA scaffolds implanted with chondrocytes subcutaneously in nude mice for seven days to evaluate the fibrocartilage formation ability of the construct. Histology results showed fibrocartilaginous neotissue generation between meniscus discs, demonstrating the feasibility of chondrocytes as cell seeds for meniscus tissue engineering. Moreover, significantly more cartilaginous tissue and complete healing of the meniscus defects was observed in a platelet-rich plasma- (PRP-) treated hyaluronic acid/PCL scaffold seeded with autologous chondrocytes in a total meniscectomy sheep model; the author believed that seeding of the scaffolds with autologous chondrocytes provided additional benefits for fibrocartilaginous tissue repair [64, 65]. However, harvesting autologous cells could cause additional trauma to patients. Weinand et al. [66] compared the repair capacity of autologous and allogeneic cells for meniscus lesions and found that the remediation results between the two cell types showed no significant difference. This experiment demonstrated the essentially equal potential of autologous and allogeneic chondrocytes for meniscus tissue engineering and will likely broaden the possible cell sources for cartilaginous tissue engineering if ethical issues can be resolved. Extensive research on chondrocytes and their successful application in cartilage repair will be beneficial for their application in meniscus tissue engineering.

## 5. Synoviocytes

As the most abundant tissue in the knee joint, the synovial membrane can affect the development of osteoarthritis by producing microRNAs and other factors [67]. Synoviocytes are considered a resource for meniscus tissue engineering due to their chondrogenic behavior. Studies have shown that synoviocytes can form a meniscus-like matrix in vitro, and FGF promotes collagen II formation and a chondrocytic cell phenotype [68, 69]. Fox et al. [70] investigated the feasibility of fibroblast cells derived from synovial tissue to generate a meniscus construct in vitro. Equine synoviocytes were seeded onto a PGA/PLLA scaffold and cultured with growth factors in a rotating bioreactor. Reverse transcription polymerase chain reaction (RT-PCR) results indicated that fibroblast cells exhibit fibrochondral characteristics, and growth factors improve the expression of collagen II and GAGs. As previously mentioned, bioreactors, which provide mechanical stimulation, have a positive effect on cell differentiation, cell viability, ECM production, and compressive biomechanical properties. As research showed culturing a synoviocyte-seeded PGA scaffold in a rotating bioreactor resulted in much better cell and matrix characteristics than when cultured in static conditions [71]. Furthermore, the research has shown that synoviocytes derived from osteoarthritic joints can produce meniscal fibrocartilage ECM components at levels similar to those of normal synoviocytes [72]. These achievements demonstrate the feasibility of synoviocytes as a potential cell source for meniscus tissue engineering.

## 6. Stem Cells

Stem cells exhibit features of multidirectional differentiation. Stem cells can be divided into embryonic stem cells and adult stem cells. The latter, such as bone marrow mesenchymal stem cells (BMSC) and adipose-derived stem cells (ADSC), have been a focus of research due to their wide distribution and species diversity [73, 74]. Studies have shown that stem cells can differentiate into cartilaginous cells with abundant matrix; therefore, stem cells can be used as cell seeds for meniscus tissue engineering [75, 76]. Yamasaki et al. [77] built a construct similar to a native meniscus using rat BMSC and a decellularized meniscus scaffold. Nerurkar et al. [78] and Baker et al. [44] built a meniscus substitute with bovine BMSC and aligned PCL electrospun scaffolds and found that dynamic culture conditions and mechanical stimulation were beneficial for stem cells infiltration and proliferation, collagen deposition, gene expression, and reinforcing mechanical properties. The ECM generated in the structure was sufficient and resembled the collagen and GAG content in the native meniscus. It is noteworthy that the duration of preculture may play a role in BMSC responding to the mechanical stimulation, because the condition of preculture can provide a sufficient time for cell colonization and ECM deposition. In addition, human adult BMSC were also investigated to generate a meniscus substitute. The results showed that BMSC improved the tensile strength of collagen scaffold, and mechanical stimulation enhanced the mechanical properties of the structure [34, 48].

Many MSCs exist in synovial membranes and can be induced to differentiate into chondrocytes under appropriate culture conditions [79, 80]. Sakimura et al. [81] found synergistic effects of TGF-*β*1 and Insulin-like Growth Factor-1 (IGF-1) on synovial MSC in cartilaginous differentiation and GAG production for repairing meniscus defects. Some researchers believe that MSCs in synovial tissue can migrate to damaged areas to promote restoration, just like the scaffold loaded with CTGF and TGF-*β*3 can regenerate a meniscus via stem cell recruiting [37]. Injections of MSC derived from the synovial membrane have been used to repair meniscus defects in rat, rabbit, and pig models, resulting in effective new fibrocartilage generation [82–84]. Due to their quantity, ease of acquisition and multidirectional differentiation, synovial MSC may represent good prospects in future meniscus tissue engineering.

Direct injection of stem cells into the knee joint for the treatment of meniscus defects and osteoarthritis has been assessed in clinical trials [85]. However, tissue engineering is a better method to infuse a large number of stem cells. Not only does a scaffold provide a specific space for cell attachment and proliferation to maintain high levels of stem cells in a particular area, but also loading growth factors induce stem cell homing to the defect to improve repair and regeneration results.

## 7. Cell Coculture

According to previous research, with a tissue-engineered meniscus based on a single cell type, it is difficult to copy microstructural features of the native meniscus. Cell coculture, culturing two cell types, is a strategy used to improve the biochemical and biomechanical properties of a tissue-engineered meniscus through cellular interactions and mitigates the need for cell expansion [86]. There are two types of cell coculture systems: direct and indirect. The former is a mixed cell culture, which works through direct cell interaction, while the latter involves separating the cell culture in one system and works through the transfer of growth factors; this is also known as paracrine secretion.

Research has shown that collagen dominates the tensile response while GAG is important for compressive properties [43]. One study found that meniscus fibrochondrocytes alone were not able to produce enough collagen type II and GAG, while pure chondrocytes were unable to deposit sufficient collagen type I to sustain tensile strength. Could coculturing two types of somatic cells have a synergistic effect? Gunja and Athanasiou [87] built a construct by coculturing meniscus cells and chondrocytes on PLLA scaffolds and found that coculturing not only increased ECM secretion but also generated a meniscus structure with better mechanical properties. The compressive properties in cell-seeded constructs approached those of the native meniscus. However, much work needs to be done which enhances the tensile properties in the cell-seeded constructs towards those of the native tissue.

MSCs have the potential to differentiate along the chondrogenic pathway in appropriate conditions, as well as deliver trophic effects to differentiated cells [88–90]. In a coculture system, somatic cells provide inductive stimulation for stem cell differentiation, while stem cells supply various growth factors to maintain cellular phenotype and regulate cell proliferation. Previous studies reported that coculture of MSC with chondrocytes enhances ECM production and inhibits hypertrophy [91], so why not with meniscus cells? Cui et al. [92] cocultured meniscus cells with MSCs at different ratios and found that coculturing not only promoted levels of collagen type I and GAG production but also prevented cell hypertrophy, with meniscus cells and MSC at a 75 : 25 ratio showing the best results. The studies also found that coculturing meniscus cells isolated from osteoarthritic patients with BMSC in a pallet contribute to similar results as normal meniscus cells [93, 94]. These studies strengthen the combined application of meniscus cells and MSCs as a cell source for meniscus tissue engineering. Tan et al. [95] cocultured meniscus cells with synovium-derived stem cells on small intestine submucosa to engineer a meniscus substitute construct and found that this coculture-based tissue construct exhibited higher GAG and collagen II levels, resulting in a concomitant increase in mechanical properties. MSCs derived from synovial tissue secrete IGF-1 with anti-inflammatory effects, which is beneficial in preventing osteoarthritis [96]. Thus, cell coculture not only enriches the choice of cell seeds but also serves as an effective method to maintain cellular phenotype and improve repair effects and may represent a promising strategy for meniscus tissue engineering.

## 8. Zonal Recapitulation

Compositional differences in the meniscus inner and outer regions make it difficult to fabricate a bionic meniscus. To generate a tissue-engineered meniscus successfully, mimicking its complex internal architecture is important. The heterogeneity of cell types and matrix components in the inner and outer regions makes it nearly impossible for meniscus reconstruction and complete regeneration via tissue engineering methods, especially when relying on a method to build a construct with a single cell type and scaffold. Hence, the idea of seeding different cell types on different regions of a scaffold to create a biomimetic substitute in both structure and content has been developed. This strategy simultaneously creates an indirect coculture system when effectively applying the cells' different physiological characteristics. Mandal et al. [33] have designed a multilayer meniscus scaffold fabricated with silk to mimic native meniscus structure; human articular chondrocytes and dermal fibroblast cells were seeded at the periphery and center of the scaffold, respectively. Scanning electron microscopic (SEM) results reveled that the top two layers showed circular pores, while the bottom third layer had laminar channels. After culturing for four weeks in vitro, the results showed that the cell-seeded construct improved mechanical properties, increased cell populations, enhanced ECM deposition, and maintained a chondrocytic phenotype. Thus, the author suggested that a construct with multiple layers and porosity can act as an effective medium to direct cell orientation and neotissue formation, resembling the structural and mechanical anisotropy of the native meniscus. Higashioka et al. [97] used agar without cell adhesion to build an anisotropic graft with zonal variations; the inner one-third was seeded with 100% chondrocytes and the outer region was generated by coculturing chondrocytes with meniscus cells. The two zones of the structure integrated well and exhibited significantly different mechanical and biochemical properties after culturing. This is a case that combined zonal recapitulation with cell coculture. With the application of 3D printing technology, it may be more conducive to regenerate a construct that is more biomimetic of the native meniscus. Zonal recapitulation is a more suitable method to mimic the complex internal architecture of the meniscus and may be a better cell application strategy for meniscus tissue engineering.

## 9. Scaffold-Free Tissue Engineering

In “traditional” meniscus tissue engineering, the scaffold is an important factor. Biodegradability, biocompatibility, and certain mechanical strength are necessary for a scaffold [39]. However, it is difficult to copy the complex internal structure of the meniscus with the technologies available today. Some researchers have proposed generating a new meniscus relying on self-assembling cells in a specific artificial environment, simulating the tissue generation process; this is also referred to as scaffold-free culture [98]. High-density cells form a tissue through close interactions in a specific environment, which not only reduces the obstruction of the scaffold but also is more conducive to crosslinking collagen fibers in the ECM to promote mechanical strength [99]. This method can also be divided into single cell type self-assembly and coculture self-assembly.

## 10. Single Cell Type Self-Assembly

Single cell type self-assembly is mostly used to regenerate the inner part of the meniscus, in which fibrochondrocytes represent the main cell type. A series of experiments were conducted to explore the possibility of generating a neotissue via cell self-assembly. Ballard et al. [100] compared the properties of bioscaffolds generated by meniscus fibrochondrocytes and synoviocytes and determined they had similar collagen and GAG content. He also found that treating synovial neotissue with chondrogenic growth factors (bFGF, TGF-*β*1, and IGF-1) or mechanical stimulation showed greater fibrocartilage-like matrix content and better biomechanical properties [101]. In addition, he demonstrated the fibrochondrogenic potential of synoviocytes from osteoarthritic joints, making them a potential cell source for meniscal tissue engineering [72]. These studies confirm the feasibility of a synovial bioscaffold as a replacement therapy for meniscus defects. Moreover, Moriguchi et al. [102] used an allogeneic synovial MSC bioscaffold to repair swine meniscus defects. The defects implanted with the bioscaffold were consistently repaired by fibrocartilaginous tissue with good tissue integration and cartilage protection after six months. These results suggest that a scaffold-free tissue-engineered construct could represent a promising cell-based implant to repair meniscus lesions.

## 11. Cells Coculture Self-Assembly

Cell coculture not only offers more options to generate a meniscus construct but also promotes biochemical and biomechanical properties of the tissue-engineered meniscus. Hoben et al. [103] compared constructs with self-assembling chondrocytes, meniscus fibrochondrocytes, and cocultures of fibrochondrocytes and chondrocytes and found that the coculture resulted in a mixed collagen I and collagen II matrix similar to the native meniscus. To achieve a scaffold-free construct with better mechanical properties mimicking the native meniscus, Huey and Athanasiou [104] combined the self-assembly process with the catabolic enzyme chondroitinase-ABC and TGF-*β*1 and resulted in a mature neotissue with a higher radial and compressive modulus. This study revealed that self-assembly approach can produce a tissue-engineered construct that is similar to the biochemical and biomechanical characteristics of native meniscus. Hadidi and Athanasiou [105] applied the phospholipid lysophosphatidic acid (LPA) to enhance the mechanical properties of a construct generated by chondrocytes and meniscus cells via a self-assembling process. Moreover, this research further revealed that LPA mainly depended on inducing cytoskeletal reorganization and cell-matrix traction to enhance the mechanical properties. He also investigated the appropriate number of cells to create a scaffold-free structure for meniscus tissue engineering and found that coculturing chondrocytes and meniscus cells at a ratio of 1 : 1 with lower seeding density resulted in beneficial effects on self-assembling fibrocartilage [106]. These studies have shown the feasibility of generating an effective substitute without a scaffold, and the results demonstrated favorable biomechanical properties of the neotissue. From these achievements, we conclude that cells are the most important factor for scaffold-free culturing, and obtaining a sufficient number of cells is the key for applying this technology.

In the future, it will be vital to solve the meniscus regeneration problem, regardless of how the meniscus is created, especially the injuries in the white-white areas of the meniscus, because the role vessels play in maintaining the meniscus in this areas is inadequate. This dilemma might be addressed by the following approaches. In the one side, creating some vascular channels to the scaffolds or direct injection of some stem cells may enhance the meniscus regeneration to some extent; on the other side, loading some growth factors or PRP to the scaffolds may also be beneficial to form a tissue-engineered meniscus. However, some drawbacks exist in the application of constructs. For scaffolds with a single cell type or cell coculture, the mechanical properties are sufficient if the construct is only needed for avascular region regeneration, but it will be difficult for fixation and ideal results, whereas if the construct is needed for whole regeneration, it will be difficult to satisfy the required radial tensile strength. For zonal recapitulation, the mechanical difference between the inner and outer regions is large, and the transition zone will tear easily. For scaffold-free culturing, improving the mechanical properties remains important.

Considering all the current advances, we can envisage an ideal tissue-engineered meniscus in the future: regarding the meniscus internal structure as a blueprint and using the meniscus ECM/cells mixture as material for 3D printing technology, it will be possible to build a tissue-engineered meniscus with zonal heterogeneity, while growth factors within the construct will not only promote cell proliferation and maintain cellular phenotype but also induce recruiting of stem cells in vivo to promote meniscus regeneration. However, for a cell-scaffold construct in vivo, cell supply issues and how to mobilize the migration of stem cells are problems that still need to be resolved.

## 12. Conclusions

Meniscus tissue engineering is a main focus but remains difficult. It will be essential to create a structure mimicking the architectural and mechanical properties of the native meniscus. Because the natural complex internal architecture is still not absolutely clear, a method based on zonal recapitulation may be more promising with the further development of 3D printing technology. However, scaffold-free cell self-assembly methods to generate a functional meniscus graft with good mechanical properties may be a possibility if an appropriate environment can be created. In conclusion, three main parts, scaffolds, cells and growth factors, need further clarification in order to create an ideal tissue-engineered meniscus. We still have a long way to go.

## Figures and Tables

**Table 1:** Cell application in meniscus tissue engineering.

Strategy	Cell application	Cell resource	Author
Single cell type	MC	Human (18–84 years)	Baker et al., 2009 [58]
		New Zealand white rabbits	Gunja and Athanasiou, 2010 [41]
		Allogeneic rabbit cell	Kang et al., 2006 [32]
		New Zealand white rabbits	Esposito et al., 2013 [62]
	AC	Swine	Yoo et al., 2011 [63]
		Autologous sheep chondrocytes	Kon et al., 2008 [64]
		Autologous and allogenic swine chondrocytes	Weinand et al., 2006 [66]
	SMC	Adult equine	Fox et al., 2010 [70]
		Canine	Warnock et al., 2014 [71]
	SC	Rat bone marrow	Yamasaki et al., 2005 [77]
		Femoral and tibial bone marrow of calves	Nerurkar et al., 2011 [78]
		Femoral and tibial bone marrow of calves	Baker et al., 2011 [44]
		Human iliac crest (27–55 years)	Petri et al., 2012 [48]
		Adult human synovial membranes	Sakimura et al., 2006 [81]

Cell coculture	MC and AC	Knee joint of rabbit	Gunja and Athanasiou, 2009 [87]
	MC and MSCs	Human meniscus and human MSCs	Cui et al., 2012 [92]
	MC and BMSC	Human meniscus and iliac crest of patients	Diao et al., 2013 [91]
	MC and BMSC	Human meniscus and human MSCs	Matthies et al., 2013 [93]
	MC and SMSC	The knees of pigs	Tan et al., 2010 [95]

Zonal recapitulation	AC and FBC	Human donors (<35 years)	Mandal et al., 2011 [33]
	AC and MC	Stifle joint of calves	Higashioka et al., 2014 [97]

Scaffold-free	SMC	Synovial villi from canine with stifle osteoarthritis	Warnock et al., 2014 [72]
	SMSC	Porcine synovial membranes	Moriguchi et al., 2013 [102]
	MC and AC	Knee joints of calves	Huey and Athanasiou, 2011 [104]
	MC and AC	Knee joints of calves	Hadidi et al., 2015 [106]

MC: meniscus cell, AC: articular chondrocyte, MSC: mesenchymal stem cells, BMSC: bone marrow mesenchymal stem cells, SMC: synovial membrane cell, SC: stem cell, SMSC: synovial membrane mesenchymal stem cells, FBC: fibroblast cells, LPA: phospholipid lysophosphatidic acid, FBS: fetal bovine serum, and ADSC: adipose-derived stem cells.
